# Monitoring neovascularization and integration of decellularized human scaffolds using photoacoustic imaging

**DOI:** 10.1016/j.pacs.2019.01.001

**Published:** 2019-01-08

**Authors:** Olumide Ogunlade, Jasmine O.Y. Ho, Tammy L. Kalber, Robert E. Hynds, Edward Zhang, Sam M. Janes, Martin A. Birchall, Colin R. Butler, Paul Beard

**Affiliations:** a Department of Medical Physics & Biomedical Engineering, University College London, London, UK; b UCL Ear Institute, University College London, UK; c UCL Centre for Advanced Biomedical Imaging, Division of Medicine, University College London, UK; d Lungs for Living Research Centre, UCL Respiratory, University College London, London, UK

**Keywords:** Angiogenesis, Tissue engineering, Trachea, Transplantation, Vascularization, Photoacoustic imaging

## Abstract

Tissue engineering is a branch of regenerative medicine that aims to manipulate cells and scaffolds to create bioartificial tissues and organs for patients. A major challenge lies in monitoring the blood supply to the new tissue following transplantation: the integration and neovascularization of scaffolds *in vivo* is critical to their functionality. Photoacoustic imaging (PAI) is a laser-generated ultrasound-based technique that is particularly well suited to visualising microvasculature due to the high optical absorption of haemoglobin. Here, we describe an early proof-of-concept study in which PAI in widefield tomography mode is used to image biological, decellularized human tracheal scaffolds. We found that PAI allowed the longitudinal tracking of scaffold integration into subcutaneous murine tissue with high spatial resolution at depth over an extended period of time. The results of the study were consistent with post-imaging histological analyses, demonstrating that PAI can be used to non-invasively monitor the extent of vascularization in biological tissue-engineered scaffolds. We propose that this technique may be a valuable tool for studies designed to test interventions aimed at improving the speed and extent of scaffold neovascularization in tissue engineering. With technological refinement, it could also permit *in vivo* monitoring of revascularization in patients, for example to determine timing of heterotopic graft transfer.

## 1 Introduction

The field of tissue engineering aims to regenerate tissues or whole organs for clinical transplantation and fuses aspects of materials science, biomedical engineering and cell biology. An important lesson from early clinical translation of tissue-engineered products in various settings has been the importance of scaffold integration for long-term graft survival. Several processes are involved in the integration of the scaffold with recipient tissue, but one important aspect is the establishment of a vascular network within the graft as the requirement for nutrients and oxygen often cannot be met by simple diffusion. The ability to monitor angiogenesis, the process of new blood vessel formation from pre-existing vasculature [1,2], in implanted scaffolds in real-time *in vivo* would facilitate comparisons of vascularization in different scaffold materials and assist in the development of therapeutic strategies that promote vascularization [3,4]. However, monitoring vascularization in bioengineered scaffolds is currently challenging [5]. As such, the development of new methods to investigate angiogenesis in tissue-engineered scaffolds could lead to improved therapeutic outcomes for patients who require bioengineered tissues and organs [6].

The techniques currently used for preclinical *in vivo* monitoring of angiogenesis include magnetic resonance imaging (MRI), ultrasound (US), micro-computed tomography (microCT), positron emission tomography (PET), single-photon emission computed tomography (SPECT), optical microscopy and optical coherence tomography (OCT) [[7], [8], [9], [10]]. The limitations of these techniques include the need for exogenous contrast agents or radiotracers for visualizing small vessels in MRI, PET, SPECT and ultrasound and the use of ionizing radiation in microCT [7,9]. Optical imaging techniques such as confocal, multi-photon microscopy or OCT can provide high resolution images of the microvasculature without the need for ionizing radiation [[11], [12], [13]] but the penetration depth that can be obtained is limited to approximately 1 mm due to the strong optical scattering exhibited by tissue [14]. Photoacoustic imaging (PAI) is an emerging hybrid imaging modality that offers the prospect of overcoming these limitations [[15], [16], [17]]. It is based upon the generation of broadband ultrasound waves by the absorption of low energy laser pulses by tissue chromophores. These waves are then detected at the tissue surface and used to reconstruct a 3D image of the internal tissue structure. This approach offers several advantages. Since acoustic waves are scattered much less than photons in tissue, it avoids the penetration depth limitations of the purely optical imaging techniques mentioned above. In addition, contrast is defined primarily by optical absorption. This makes PAI particularly well suited to visualizing the microvasculature without the need for exogenous contrast agents due to the strong optical absorption of hemoglobin [18]. Several preclinical studies have exploited the high microvascular contrast provided by PAI to non-invasively visualize angiogenesis in simple, synthetic, non-biological poly(lactic-co-glycolic acid) (PLGA) scaffolds implanted subcutaneously in the rodent ear or flank [19,20]. However, the utility of PAI for determining the integration and vascularization of human biological scaffolds has not yet been investigated. Biological scaffolds have greater potential to be clinically translated as bioartificial tissues and organs than synthetic scaffolds because of their near-native tissue architecture and biocompatibility in the recipient [21].

In the current work, a proof-of-concept study was undertaken in which the integration and neovascularization of a complex composite biological tissue scaffold – decellularized human trachea – was monitored longitudinally over a 15-week period. This decellularized tissue, consisting of layers of cartilage, fibrous intercartilaginous zones, muscle and mucosa, was implanted subcutaneously into murine flank and PAI in tomography mode was used to visualize neovascularization. This enabled the scaffold to be visualized at greater depths than optical microscopy techniques, including optical resolution photoacoustic microscopy (OR-PAM), where the imaging depth is limited to approximately 1 mm by tissue optical scattering [22].

## 2 Materials and methods

### 2.1 Scaffold preparation

Human tracheae were obtained from cadaveric donors aged between 30–80 years by the National Health Service Blood and Transplant (NHSBT) tissue retrieval team. Ethical approval was granted by the National Research Ethics Committee (REC reference 11/LO/1522). Tracheae were retrieved within the first 48 h post-mortem and removed in their entirety from cricoid to carina. After retrieval, donor tracheae were rinsed in 1 L 0.9% normal saline and surrounding tissue was dissected away. The tracheae were immersed in 20% chlorhexidine solution for 5 min followed by a further three washes in 0.9% saline.

Intact tracheae were processed using the detergent-enzymatic decellularization method (DEM) as described by Conconi et al. [23]. Briefly, tracheae were subjected to 25 cycles of distilled water for 72 h at 4 °C, 4% sodium deoxycholate (Sigma) for 4 h and 2000 kU DNase-I in 1 M sodium chloride (Sigma) for 3 h. They were continuously rotated on a roller throughout the decellularization process. These were fashioned into 0.5–1.0 cm width, full-thickness tissue graft and fiducial sutures (5/0 Prolene) were inserted into the scaffold. After decellularization, scaffolds were gamma irradiated (10,000 Gy) and were stored at 4 °C in PBS until use. One scaffold was injected with 33 ng VEGF (Sigma) in 100 μl PBS at the time of implantation.

### 2.2 Subcutaneous implantation model

**Fig. 1:**
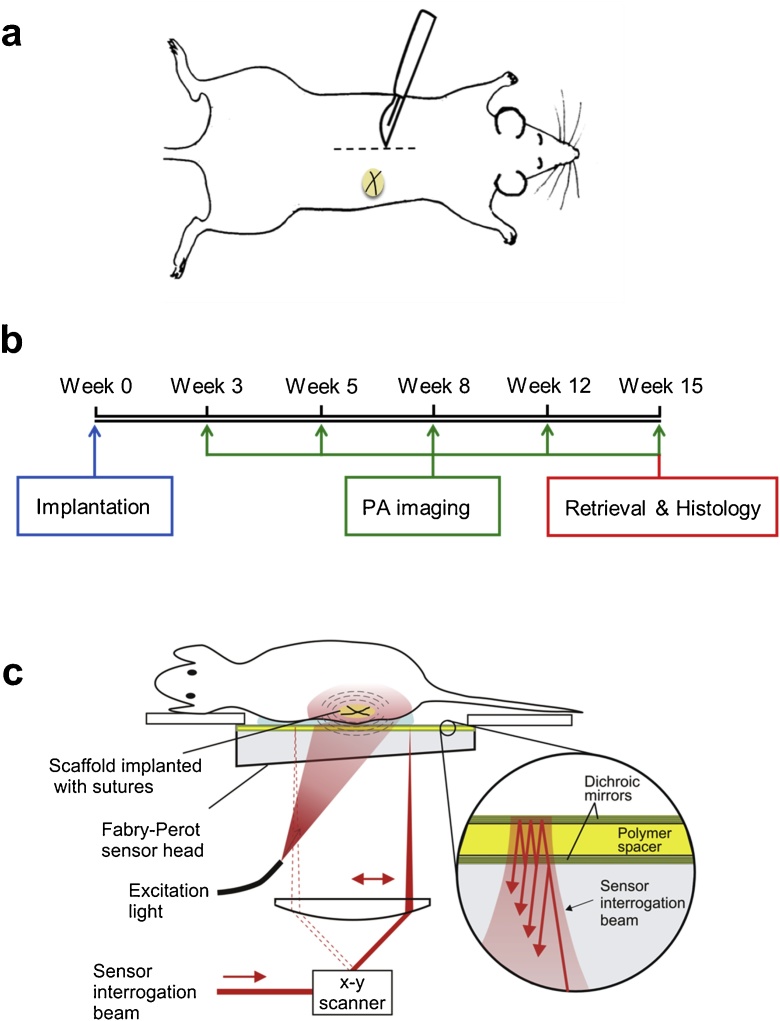
Photoacoustic imaging to monitor neovascularization in biological tissue-engineered scaffolds. (a) A dorsal incision was created on the flank of a mouse and a scaffold, containing fiducial sutures, was implanted subcutaneously within a pocket fashioned on the flank, away from the incision line. (b) Experimental timelines showing the scaffold implantation, photoacoustic (PA) imaging time points and retrieval of the implanted scaffold. (c) A schematic showing the PA tomographic imaging setup. A large diameter pulsed laser beam illuminates the tissue volume containing the implanted scaffold, generating photoacoustic waves. The photoacoustic waves are detected by the all-optical Fabry-Perot (FP) ultrasound sensor head. The FP sensor is read-out by optically scanning a focused sensor interrogation laser beam across its surface, recording the photoacoustic signals at each scan point.

All animal studies were approved by the University College London Biological Services Ethical Review Committee and licensed under the UK Home Office regulations and the Guidance for the Operation of Animals (Scientific Procedures) Act 1986 (Home Office, London, United Kingdom). Adult CD1 mice (n = 6) were used for subcutaneous implantation of scaffolds. Mice were anaesthetized with a 2–5% isoflorane:oxygen gas mix for induction and maintenance. Under aseptic conditions, the dorsum of each mouse was shaved and chlorhexidine solution applied. A 5 mm transverse dorsal incision was made to raise a subcutaneous pocket for implantation of the scaffold (Fig. 1a). This was done so that the scaffold was implanted away from the surgical incision site, ensuring that there is as little influence as possible from angiogenesis resulting from superficial skin wound healing. A single prepared tracheal scaffold, 7 mm in diameter, with an accompanying nylon marking suture fashioned as a cross, was inserted into the pocket. The suture is optically absorbing and thus provides photoacoustic contrast enabling it to be used as an identification marker during the imaging process. The pocket was closed with buried 4/0 Vicryl interrupted sutures. The scaffolds implanted in (n = 3) mice were imaged longitudinally at five set time points, as described in Section 2.3, for a total of 15 weeks. After 15 weeks, the scaffolds were retrieved *en bloc* for histological analyses. A timeline of the study is shown is shown in Fig. 1b.

### 2.3 Photoacoustic Imaging (PAI)

Photoacoustic signals were generated and detected using an all-optical preclinical photoacoustic scanner which operates in tomography mode. This mode of PAI uses a wide-field excitation beam to irradiate a relatively large volume of tissue [15]. It provides greater penetration depth (mm-cm) than the sub-mm depths provided by optical resolution photoacoustic microscopy (OR-PAM) [22] previously used to characterize synthetic scaffolds [19]. A detailed description of the design and operation of the scanner, which is based on a Fabry-Perot (FP) polymer film ultrasound sensor, has been reported previously [24,25], but a brief description is given here, following the schematic given in Fig. 1c.

The scanner employs a tunable optical parametric oscillator (OPO) based laser system (Quanta Ray Pro-270/premiScan, Newport Spectra Physics/GWU Lasertechnik) as the excitation source. This system provides 7 ns laser pulses at a pulse repetition frequency of 50 Hz and can be tuned over the range 410 nm–2100 nm. The output of the laser is coupled into a multimode optical fibre. The distal end of the fibre was positioned so as to irradiate the skin surface with a 2 cm diameter beam which is transmitted through the FP sensor; the latter is possible because the sensor is designed to be transparent to wavelengths between 590 nm and 1200 nm. The absorption of the excitation light by the tissue generates photoacoustic waves which are detected by the sensor on to which the mouse was placed. The sensor itself is a thin film structure comprising two dichroic mirrors, separated by a 40 μm thick polymer film spacer, thus forming a Fabry Perot interferometer. Photoacoustic waves incident on the sensor modulate the optical thickness of the polymer spacer. This produces a corresponding modulation in the sensor reflectivity which is detected using a focused interrogation laser beam at ˜1550 nm (where the sensor mirrors are highly reflective) that is incident on the sensor. In order to map the spatial distribution of the incident photoacoustic waves, the interrogation laser beam is scanned point-by-point over the surface of the sensor. The sensor has a uniform broadband frequency response with a -3 dB bandwidth of 22 MHz and the optically defined element diameter was 60 μm. The scanner provides a maximum lateral field of view of 20 × 20 mm, a depth-dependent spatial resolution in the range 50–150 μm and a penetration depth of approximately 10 mm depending on excitation wavelength [24,25].

Before imaging, mice were anaesthetized using isoflurane in oxygen [4% (vol/vol) at a flow rate of 2 L/min for induction and 1.5% (vol/vol) at a flow rate of 1 L/min for maintenance]. A regulated heating system maintained the ambient temperature around the anaesthetized mice at 34 °C. In order to achieve acoustic coupling between the sensor and the mice, a small amount of ultrasound gel was applied to the surface of the mouse skin. In this study, the scan area was 14 mm x 14 mm with a step size of 100 μm. For each scan, approximately 20,000 waveforms were therefore acquired, each containing 640 time points and each scan took approximately 7 min to complete, limited by the repetition rate of the excitation laser. The maximum laser pulse energy used in this study was 18 mJ. The beam diameter incident on the mouse skin was 2 cm, thus the incident fluence was below 6 mJ/cm [2].

Three-dimensional photoacoustic images were reconstructed from the acquired photoacoustic signals, using a time reversal algorithm [26] with a correction for tissue acoustic attenuation implemented using a time variant filtering method [27]. The image reconstruction algorithm was implemented using k-Wave, an open source MATLAB toolbox developed at University College London for the time-domain simulation and reconstruction of photoacoustic and ultrasound wave fields (www.k-wave.org) [28]. The sound speed used in the reconstruction was selected using an autofocus approach, based on a metric of image sharpness [29]. The reconstructed images were displayed as maximum intensity projections (MIPs) using a logarithmic image intensity scale. Volume rendering of the reconstructed 3D dataset was using Amira software (FEI Visualization Sciences).

### 2.4 Histological analysis

Retrieved samples were fixed in 4% paraformaldehyde (PFA) overnight at 4 °C prior to processing for paraffin embedding. Hematoxylin and eosin (H&E) staining was performed on 5 μm thickness paraffin sections using an automated staining system (Tissue-Tek). For immunohistochemistry, paraffin sections were dewaxed using an automated protocol and antigen retrieval was performed using citrate buffer (pH 6.0). Vasculature was identified using an antibody against endomucin (Abcam; ab106100). Species-appropriate secondary antibodies were used (AlexaFluor dyes; Molecular Probes).

## 3 Results

### 3.1 Study overview

**Fig. 2:**
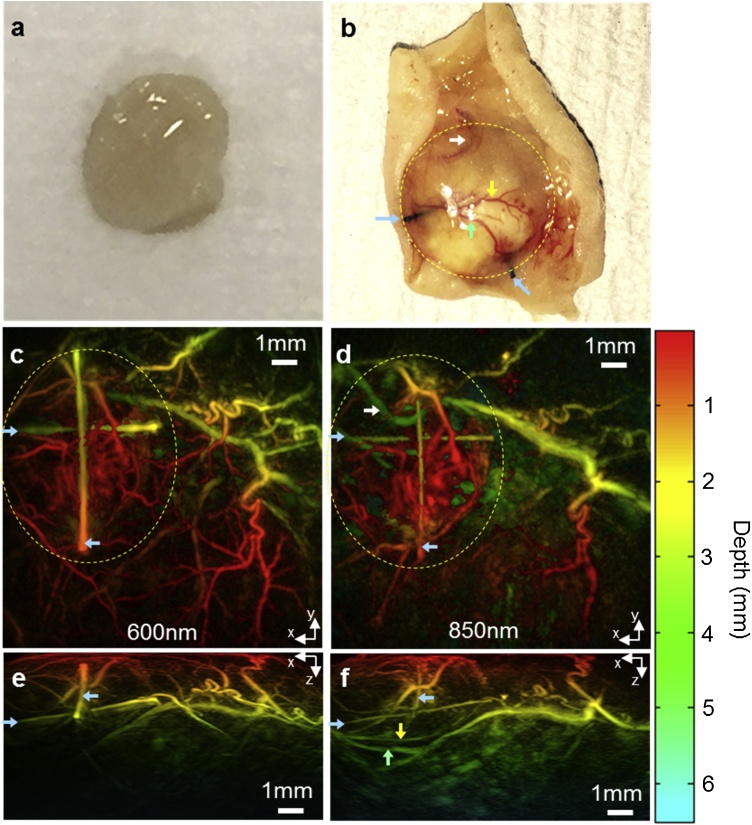
Integration of a decellularized human tracheal scaffold visualized by photoacoustic imaging. (a) Photograph of a representative acellular scaffold before implantation. (b) Photograph of a representative scaffold retrieved after 15 weeks *in vivo*. The clear translucent scaffold before implantation became integrated with the existing murine skin vasculature, indicated with green and yellow arrows. *In vivo* photoacoustic (PA) images of the same scaffold were acquired at 600 nm and 850 nm before retrieval at 15 weeks and are shown as horizontal and vertical maximum intensity projections (MIPs) (c–f). Horizontal x–y MIPs at (c) 600 nm and (d) 850 nm excitation wavelengths, respectively. The yellow dotted ellipses show the region where neo-angiogenesis occurs around the scaffold. The depth of the vessels imaged can be referenced to the color scale on right of the image, with red being most superficial and green to turquois showing vessels deeper within the tissue. The cross shaped sutures inserted into the scaffold to act as fiducial markers can be seen (indicated by blue arrows throughout). Vertical x–z MIPs acquired at (e) 600 nm and (f) 850 nm excitation wavelengths, respectively. The improved imaging depth at 850 nm, compared to 600 nm, is shown by the visibility of deeper lying vessels (yellow and green arrows) that are not visible at 600 nm (e vs f). An animated volume rendered representation of the data acquired at 850 nm is available online. In the volume rendered animation, the suture markers within the scaffolds have been false coloured blue, to aid identification of the scaffold. The scaffold itself does not provide PA contrast, and thus appears as a void. The vasculature that is false colored red is located on the underside of the scaffold and is connected to the skin vasculature. (MP4 video, 1.1MB).

Donor human tracheae were decellularized before subcutaneous implantation into the dorsal flank of mice (Fig. 1a). The incisions healed without complications in 5–7 days and there were no wound or implant complications during the 15 weeks of the study. The integration of the tissue-engineered scaffold was monitored by acquiring photoacoustic images at five time points over the course of 15 weeks (Fig. 1b). At the end of the study, the decellularized scaffolds appeared well-integrated with host tissue macroscopically (Fig. 2b). Larger mature vessels overlying the scaffold were visible. All mice tolerated the study well and recovered uneventfully from anesthesia during repeated imaging.

### 3.2 Optimization of photoacoustic imaging for visualization of tissue-engineered scaffolds

To identify the optimum imaging parameters and provide an initial assessment of the ability of the scanner to visualize neovascularization around the implanted scaffolds, a set of *in vivo* photoacoustic images was initially acquired at multiple wavelengths between 600 nm and 850 nm at a single time point (15 weeks). Fig. 2c–f show a subset of these images acquired at 600 nm and 850 nm as coronal x–y depth color-coded maximum intensity projections (MIP). The sutures acted as fiducial markers (Fig. 2b, blue arrows) enabling the scaffold to be unambiguously located in the PA images. A dense web of vasculature, due to neo-angiogenesis, is visible in the superficial region around the scaffold as indicated by the yellow dotted ellipses in Fig. 2c and d. The higher absorption of hemoglobin at 600 nm resulted in higher signal for the superficial microvasculature compared to the 850 nm image (Fig. 2c vs Fig. 2d); the signal was approximately an order of magnitude higher at the shorter wavelength, although this is not visually apparent in Fig. 2c and d as the image colour scale is mapped to depth rather than signal amplitude. However, as seen in the transverse (x–z) MIPs (Fig. 2e and f), the higher absorption results in significantly reduced imaging depth at 600nm: while the sutures indicated by the blue arrows can be seen at both 600 nm and 850 nm (Fig. 2e and f), the deeper blood vessels which are located beneath the scaffold (indicated by yellow and green arrows in Fig. 2b) are visible only at 850 nm (Fig. 2f). At 850 nm, it was possible to visualize features to a depth of approximately 6.8 mm and thus image the full thickness of the scaffold. The 3D nature of the image and the connectivity between vessels that extend from the skin vasculature to the underside of the scaffold is clearly visible in an animated volume rendered representation of the dataset (Online Video 1).

### 3.3 Longitudinal monitoring of neovascularization

**Fig. 3:**
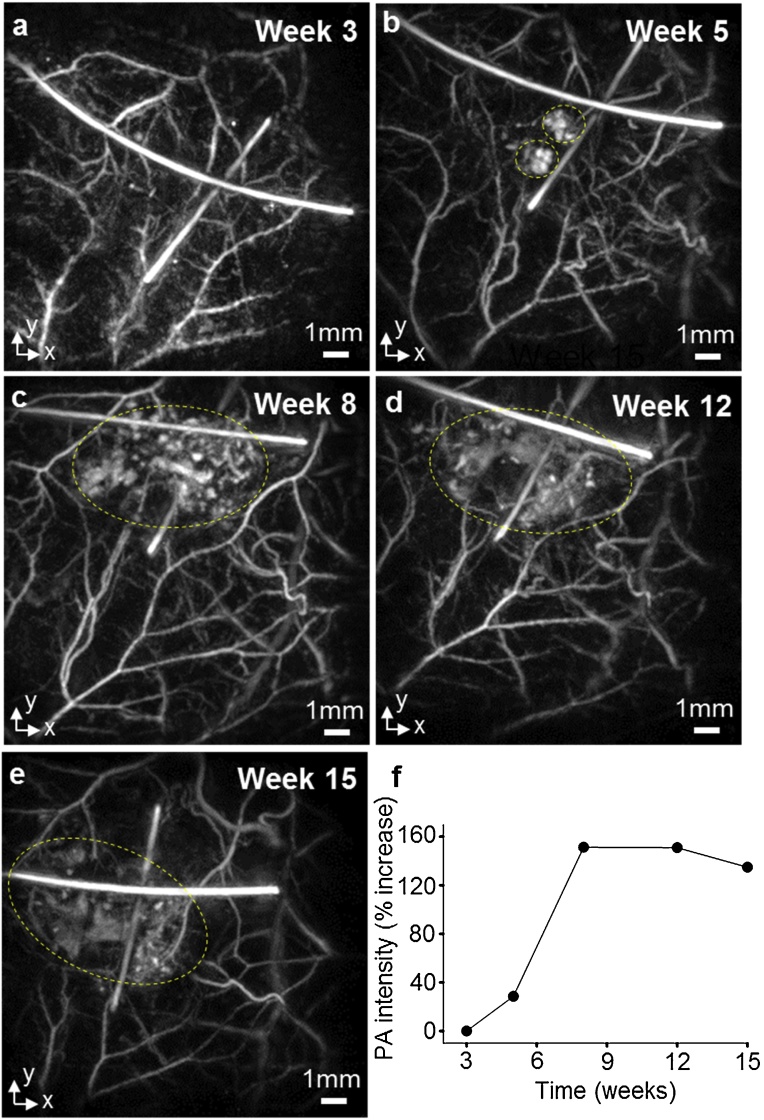
Longitudinal *in vivo* photoacoustic images showing neovascularization of a decellularized human tissue-engineered scaffold. Horizontal (x–y) maximum intensity projections (MIPs) of 3D dataset obtained in the same animal at (a) 3 weeks, (b) 5 weeks, (c) 8 weeks, (d) 12 weeks and (e) 15 weeks after implantation of the scaffold. All images were acquired at an excitation wavelength of 600 nm as this wavelength provided the highest vascular contrast. The cross shaped sutures helped to localize the scaffold at all the time points. At three weeks after implantation, only the microvasculature of the murine skin was visible around the scaffold. Beginning at 5 weeks, increased contrast around the scaffold indicated a region of neovascularization (shown encircled with yellow dashed lines). This region of neovascularization increased between week 5 and week 8 before plateauing at 12 and 15 weeks. (f) Increase in mean PA intensity of voxels within the scaffold due to neovascularization. The signal intensity at five weeks is 28% greater than at three weeks where the contrast is due to normal microvasculature in the murine skin. The increase in signal intensity is 151% at week 8, 150% at week 12, and 135% at week 15.

**Fig. 4:**
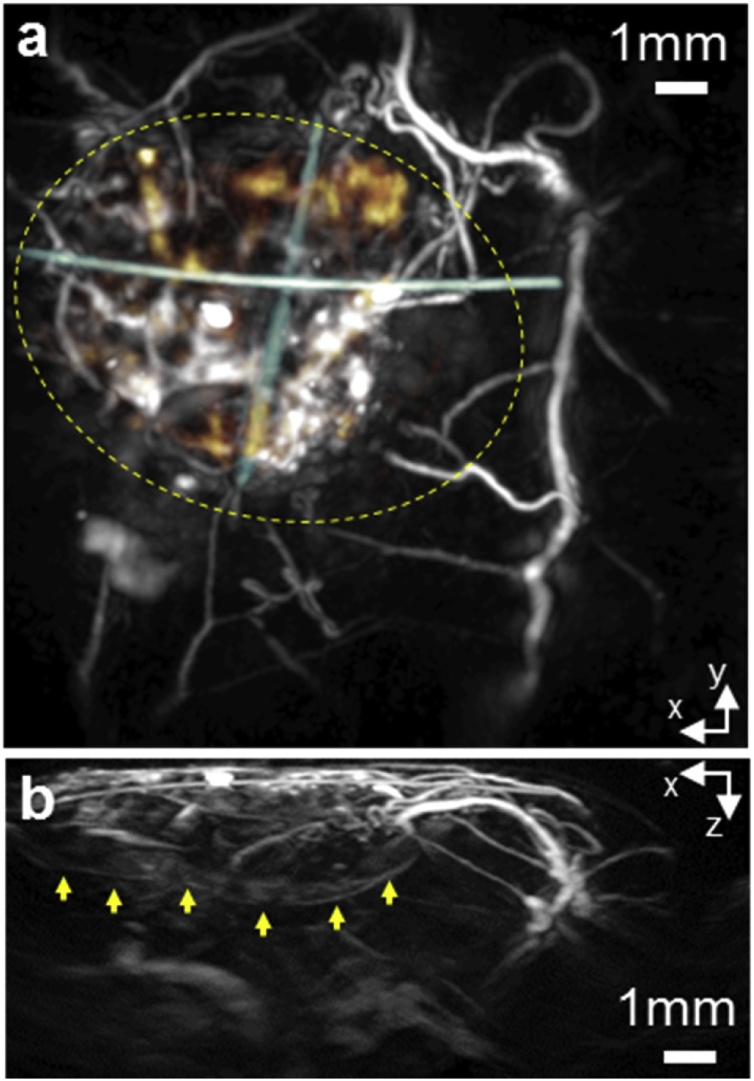
*In vivo* photoacoustic image of scaffold acquired at week 15 of longitudinal study using an excitation wavelength of 850 nm. This wavelength penetrates tissue more deeply than 600 nm enabling visualization of the neo-vasculature around the lower surface of the scaffold. (a) Horizontal (x–y) MIP with neo-vasculature beneath the scaffold false coloured yellow. The sutures indicating the location of the scaffold false are coloured blue. (b) Vertical (x–z) MIP. Yellow arrows indicate the neovascularization beneath the scaffold.

Longitudinal photoacoustic images were also acquired non-invasively at 3-, 5-, 8-, 12- and 15-week time points following scaffold implantation in order to monitor neo-vascularization. For each time point, Fig. 3 shows horizontal MIPs acquired at 600 nm as this wavelength provided the highest vascular contrast, enabling the evolution of the neo-vasculature to be visualized most clearly. The images in Fig. 3 clearly show the location of the sutures allowing unambiguous identification of the scaffold location at all time points. The image at three weeks showed only the microvasculature of the murine skin (Fig. 3a) with negligible neovascularization around the scaffold evident. Beyond this time point, however, a region of increased contrast progressively developed and expanded around the decellularized scaffold (Fig. 3b–e; indicated by the dashed ellipse), indicating neo-vascularization, before plateauing at weeks 12 and 15. To provide a quantitative estimate of neovascularisation within the scaffolds, the mean intensity of voxels that are above the noise floor in the area underneath the suture markers (Fig. 3a–e) was calculated (Fig. 3f). The noise floor was obtained by estimating the standard deviation of voxels above the mouse skin, where there is no contrast, and multiplying by a factor of three to obtain the peak value. The mean intensity of voxels within the scaffold at week 5 is 28% greater than at week 3 (Fig. 4f). The calculated increase is 151% at week 8, 150% at week 12, and 135% at week 15. The increase in mean voxel intensity corresponds to an increase in vascular contrast due to angiogenesis within the scaffold, since the contrast at week 3 is due to microvasculature of the murine skin alone. Whilst the images in Fig. 3 showed the development of the superficial vasculature with high contrast, neo-angiogenesis around the lower surface of the scaffold was not apparent due to the limited light penetration at 600 nm. However, neo-vascularization around the underside of the scaffold was observed at 850 nm due to deeper tissue penetration at this wavelength. An illustrative example of this is provided in Fig. 4 which shows horizontal (x–y) and vertical (x–z) MIPs of the same region as Fig. 3e.

### 3.4 Histological analysis

**Fig. 5:**
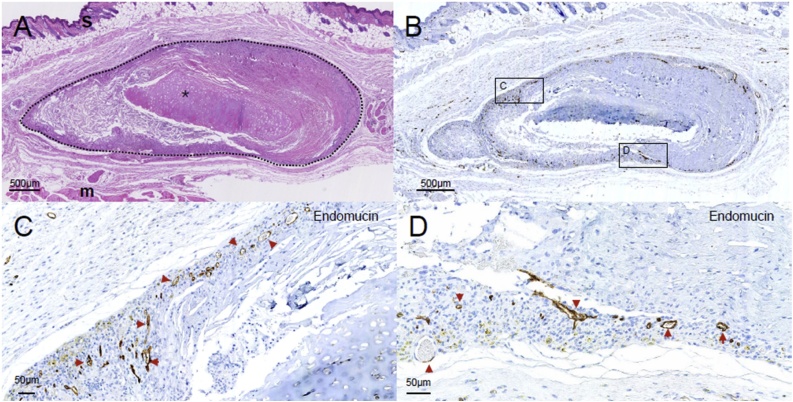
Histological analysis of decellularized human tracheal scaffolds retrieved 15 weeks after subcutaneous implantation in mice. (a) Haematoxylin and eosin (H&E) staining of scaffold (located within dotted line) with adjacent deep muscle tissue (indicated **m**) and superficial skin (indicated **s**). The scaffold contains a region of cartilaginous tissue (indicated *). Cellular infiltrates were seen around and within the non-cartilaginous region of the scaffold. (b) Endomucin immunohistochemical staining of vessels. Some artefacts from the loss of the scaffold tissue during processing can be seen. Boxed section C and D are representative ventral and dorsal regions for further magnification. (c, d) Magnified endomucin staining of the microvascular infiltration of the mucosal region of scaffold. Red arrows indicate blood vessels stained with endomucin located within the scaffold.

After scaffold retrieval at 15 weeks, hematoxylin and eosin (H&E) staining showed infiltration of the scaffold periphery with host cells (Fig. 5a). Consistent with previous observations [30], the cartilaginous portion of the scaffold did not show cellular infiltration, presumably as a result of its dense and avascular architecture (Fig. 5a). Next, we examined the presence of endomucin expression to identify the presence of endothelial cells and to confirm our PAI results showing vascular invasion both around and within the decellularized scaffolds (Figs. 5b–d). The vascular size distribution was broad with predominantly small vessels within the scaffold, consistent with neovascularization of the graft occurring through the generation of microvasculature from existing murine vascular networks in the surrounding tissue.

## 4 Discussion

While the combination of cells, scaffolds and growth factors required for regeneration in tissue-engineered grafts are well-studied, the ability of the host to revascularize transplanted grafts is a critical and poorly understood process. This is in part because neovascularization is most commonly studied at fixed time points by sectioning explanted grafts and undertaking histological analysis. Current imaging modalities for serial longitudinal observation of vascularization *in vivo* are limited in terms of their resolution, contrast and invasiveness [[7], [8], [9], [10], [11], [12], [13]] and as such have seen poor uptake in pre-clinical tissue engineering studies. The development and application of novel technologies that can image vascularization in tissue-engineered scaffolds is therefore required.

Photoacoustic imaging (PAI) has the potential to address the limitations of other imaging techniques on account of its ability to provide high contrast, label-free images of the microvasculature with high resolution and penetration depth in an entirely non-invasive manner [15,16]. Previous studies have shown success in using PAI to evaluate neovascularization of non-biological scaffolds *in vivo* [17,19,20] but the utility of PAI for imaging biological scaffolds, which are candidates for practical application in clinical studies, has not been investigated. The advantages of biological scaffolds over synthetic scaffolds lie in their innate mimicry of native tissue architecture and their biocompatibility. Of current biological scaffolds, decellularization of cadaveric donor tissue has emerged as a promising candidate for larger human tissue replacement procedures [21]. The decellularization process preserves the native ultrastructure, matrix composition and tissue architecture, thus providing an instructive microenvironment for tissue-specific cell attachment, growth and differentiation, which is difficult to replicate in *de novo* engineered scaffolds.

We were able to visualize neovascularization around a biological tissue-engineered scaffold *in vivo*, with high resolution over extended time periods without the need to perform a terminal procedure or use invasive contrast agents. The use of PAI, in tomography mode, allowed visualization of features to a depth of 6.8 mm which exceeds the depth of the implanted scaffold. This depth penetration is not achievable with OR-PAM, which has an imaging depth limitation of approximately 1 mm [22]. The use of different excitation wavelengths was also investigated to explore the trade-off between contrast and penetration depth. A wavelength of 600 nm was identified as optimal for the detection of smaller superficial vessels due to neovascularization. A longer excitation wavelength of 850 nm allowed greater depth penetration through the full thickness of the scaffold (Fig. 2f and Online Video 1) and thus visualization of neovascularization around the underside of the scaffold (Fig. 4). However, these images and the histological analysis suggest that the interior of the scaffold was largely avascular, as is typical of cartilaginous structures.

Our longitudinal study shows neovascularization of the decellularized construct over the course of fifteen weeks *in vivo*. An increase in contrast, due to neovascularization, was visible around the scaffolds after 5 weeks. This region of neovascularization increased at 8 weeks, before plateauing at 12 and 15 weeks. This is broadly consistent with the timescale observed in previous studies for the emergence of neo-vessels in tissue-engineered scaffolds implanted subcutaneously in rodents [31,32]. The extended duration of the study also takes into account any regional vasodilatory inflammatory effects which may occur and contribute to increased vascularisation during the initial stages after implantation of the scaffold. The time course of acute inflammatory response is in the order of days [33,34], hence any microvasculature observed in the region of the scaffold over the course of several weeks in this study is expected to be as a result of neo-angiogenesis rather than the consequences of local inflammation.

Histological analyses, on completion of the PAI studies, were also consistent with the location and nature of vascularization observed by PAI, with small vessels seen within and around the implanted decellularized scaffold. These findings are in line with previous work in non-biological scaffold imaging that suggest high correlation between microvascular changes shown by PAI and histological analyses [17]. Our preliminary study provides important proof-of-concept for PAI in clinically relevant tissue engineering applications and a useful, experimentally-based reference timeline for future studies, especially studies aiming to enhance the vascularization of bioengineered scaffolds by the addition of cells or pro-angiogenic factors.

In the current study, images of vascular anatomy only were acquired but PAI also has the potential to provide functional images [35] in which physiological parameters such as blood oxygen saturation [36,37] and flow velocity [38,39] can be measured. Hence, the technique has the potential to be used not only to monitor integration and neovascularization of tissue-engineered constructs, but also to provide crucial functional *in vivo* data [17,35]. Previous studies have explored the use of PAI in monitoring the vascularisation of skin grafts following burns injuries [[40], [41], [42], [43]]. We foresee a similar role for PAI in surgical procedures in where grafts are pre-vascularized in a heterotopic site: the scaffold could then be imaged *in situ* to inform decisions about the timing of its transplantation [44]. The widespread adoption of the technique may be accelerated by the miniaturization of handheld PAI devices and probes and is favored by the lower costs and high endogenous contrast of PAI compared to alternative techniques such as MRI and PET.

## 5 Conclusion

Establishing a vascular supply to tissue-engineered organs is critical to their *in vivo* viability. As such, the ability to monitor neovascularization in biological scaffolds *in vivo*, over extended periods of time, is an important step towards the standardization of protocols relating to their clinical implantation [45]. To our knowledge, this is the first study to achieve this using a decellularized human tissue scaffold. Our results suggest that photoacoustic imaging is a promising, non-invasive and non-destructive method for assessing angiogenesis in biological tissue-engineered scaffolds *in vivo*. Besides informing decision making in relation to implanted tissue-engineered grafts, this technique could generate new hypotheses relating to neo-angiogenesis in such implants, the exploration of which could, in turn, permit the testing of novel strategies to improve nutrient supply to bioengineered and other transplanted tissues and organs.

## Disclosures

The authors have no relevant financial interest in this article and no potential conflicts of interest to disclose.

## Funding

This work was supported by King’s College London and University College London Comprehensive Cancer Imaging Centre, Cancer Research UK and the Engineering and Physical Sciences Research Council (EPSRC), in association with the Medical Research Council and Department of Health, UK, and European Union project FAMOS (FP7 ICT, contract no. 317744). This work was partially undertaken at UCLH/UCL/GOSH who received a proportion of funding from the Department of Health's NIHR Biomedical Research Centre's funding scheme and the UCL Experimental Cancer Medicine Centre. Support from the MRC and ERC Advanced Grant Ref: 741149 are also acknowledged.
